# Investigating the Antibacterial Effects of Synthetic Gamma-Lactam Heterocycles on Methicillin-Resistant *Staphylococcus aureus* Strains and Assessing the Safety and Effectiveness of Lead Compound MFM514

**DOI:** 10.3390/molecules28062575

**Published:** 2023-03-12

**Authors:** Saiful Azmi Johari, Mastura Mohtar, Mohd Fazli Mohammat, Fatin Nur Ain Abdul Rashid, Muhamad Zulfaqar Bacho, Azman Mohamed, Mohamad Jemain Mohamad Ridhwan, Sharifah Aminah Syed Mohamad

**Affiliations:** 1 Bioactivity Programme, Natural Products Division, Forest Research Institute Malaysia (FRIM), Kepong 52109, Selangor, Malaysia; 2 Organic Synthesis Laboratory, Institute of Science, Universiti Teknologi MARA (UiTM), Puncak Alam Campus, Puncak Alam, Kuala Selangor 42300, Selangor, Malaysia; 3 Pharmacy Programme, Sultan Azlan Shah Allied Health Sciences College, Tanjung Rambutan 31250, Perak, Malaysia; 4 Faculty of Applied Sciences, Universiti Teknologi MARA (UiTM), Shah Alam 40450, Selangor, Malaysia

**Keywords:** gamma-lactam, antibacterial, oral anti-MRSA agent, acute toxicity, mice infection

## Abstract

Methicillin-resistant *Staphylococcus aureus* (MRSA) continues to be one of the main causes of hospital-acquired infections in all regions of the world, while linezolid is one of the only commercially available oral antibiotics available against this dangerous gram-positive pathogen. In this study, the antibacterial activity from 32 analogues of synthetic gamma-lactam heterocycles against MRSA was determined. Amongst screened analogues for the minimum inhibitory concentration (MIC) assay, compound **MFM514** displayed good inhibitory activity with MIC values of 7.8–15.6 µg/mL against 30 MRSA and 12 methicillin-sensitive *S. aureus* (MSSA) clinical isolates, while cytotoxicity evaluations displayed a mean inhibitory concentration (IC_50_) value of > 625 µg/mL, displaying a potential to becoming as a lead compound. In subsequent animal studies for **MFM514**, a single-dose oral acute toxicity test revealed an estimated mean lethal dose (LD_50_) value of <5000 mg/kg, while in the mice infection test, a mean effective dose (ED_50_) value of 29.39 mg/kg was obtained via oral administration. These results suggest that gamma-lactam carbon skeleton, particularly **MFM514**, is highly recommended to be evaluated further as a new safe and efficacious orally delivered antibacterial agent against MRSA.

## 1. Introduction

MRSA continues to be one of the main causes of hospital-acquired infections in all regions of the world [1,2]. This difficult and economically relevant pathogen have been known to display multidrug-resistance (MDR) properties towards a wide range of structurally unrelated antibiotics and antimicrobial agents [1,2]. In 2017, MRSA was inducted as a Priority 2: high-level bacteria in the first-ever WHO priority pathogen list for R&D of new antibiotics, which highlights the global unmet need for new antibiotics against infectious bacteria [3].

Following that, in 2021, the WHO has revealed that people infected with MRSA are 64% more likely to die than people with drug-sensitive infections [4]. Recently, a meta-analysis review has revealed that there was a global increase in MRSA infection between 4.6 and 170.6% during the COVID-19 pandemic [5]. In addition, *S. aureus* has been identified as the leading cause of bacterial death in 135 countries and was linked to more than 1 million deaths globally in 2019 [6].

On the other hand, there are only a handful of drugs, such as vancomycin, daptomycin and linezolid, in our dwindling armament against MRSA infections. The ever-increasing emergence of resistant MRSA strains in many parts of the world against these last-resort antibiotics seems to exacerbate the matter [1,6,7]. Hence, it is really a race against time for scientists and drug companies to find and develop new and alternative classes of antibiotics that could reduce MRSA infections worldwide.

Ecological awareness has made green chemistry into a beneficial and promising area of organic chemistry [8,9]. Green chemistry is based on the development of the simplest synthetic approach with the utilization of the least number of components while producing the least number of by-products and environmental threats [8,9]. This approach is often termed a one-pot reaction, method, or synthesis that is literally performed in the same vessel to produce biologically active molecules such as oseltamivir, baclofen and prostaglandin E [9].

In this study, 32 synthetic derivatives of gamma-lactam heterocycles were produced and tested against an MRSA and a methicillin-sensitive *S. aureus* (MSSA) via minimum inhibitory concentration (MIC) assay. As a result, a novel and microbiologically active heterocyclic molecule, designated as **MFM514**, was discovered. Following that, further in vitro and in vivo experiments were carried out to determine the inhibitory, safety and efficacy of **MFM514**.

## 2. Results

### 2.1. Synthesis of the Gamma-Lactams

In our ongoing efforts in the field of medicinal chemistry, our group has been focused on investigating the activity of small alkaloid molecules, specifically the synthetic gamma-lactam. In previous studies, we have reported on various strategies for these compounds [10,11,12,13]. With the aim of conducting biological studies, a library of gamma-lactam was established, and subsequent antimicrobial screening was performed. In order to gain insight into the structure-activity relationships (SAR) of the gamma-lactam ring template, a chemical exploration study was conducted.

**Scheme 1:**
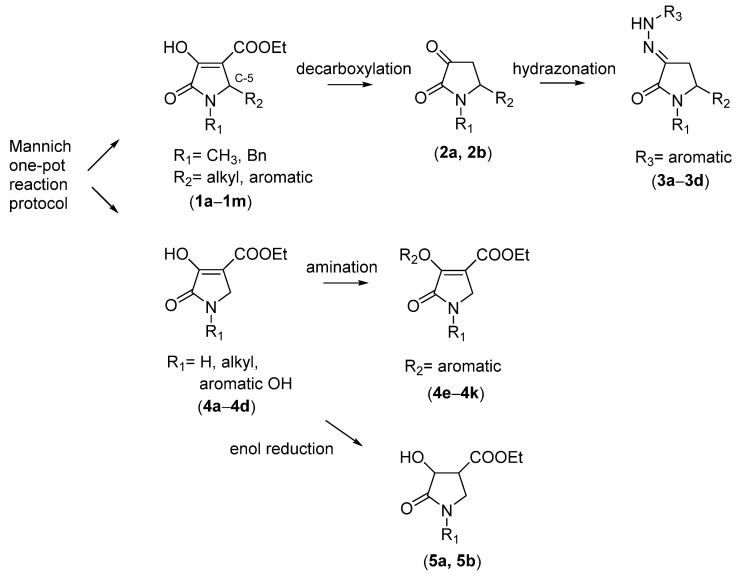
Synthesis of the gamma-lactam derivatives.

The study entailed the incorporation of various substitutions at the C-5 position, ranging from simple alkyl groups (**1a–1e**) to highly functionalized aromatic rings (**1f–1l**). The ring template was subjected to acidic conditions, resulting in the production of decarboxylated products **2a** and **2b**. These products were then reacted with hydrazine under reflux conditions to furnish various hydrazone derivatives (**3a–3d**). Additionally, a SAR study utilizing polar templates was performed, resulting in the production of derivatives **4a–4k**. The reduction of 1a and 4a through hydrogenation produced the reduced products **5a** and **5b**, respectively. The amination of **4a** and **4b** were also successfully performed, resulting in the amination products **4e–4k** (Scheme 1). All of the chemical transformations were successfully isolated with moderate to excellent yields and characterized using standard spectroscopic techniques. The full listing of the synthesized analogues of the gamma-lactam is displayed in Table 1.

### 2.2. Results of Antibacterial Test

As listed in Table 2, only **MFM514** exhibited a low MIC value of 15.6 µg/mL and 31.3 µg/mL against ATCC 33591 (MRSA) and ATCC 25923 (MSSA), respectively. On the other hand, compound1e exhibited higher MIC values of 125 µg/mL and 250 µg/mL against both isolates. Although compounds that exhibited MIC values < 64 µg/mL are considered active, only compounds that displayed MIC values < 10 µg/mL might be considered of interest to the pharmaceutical industries [14].

Following that, we evaluated **MFM514** further against additional 41 *S. aureus* clinical isolates to confirm its good inhibitory activity. As displayed in Table 3, **MFM514** showed a similar MIC value of 15.6 µg/mL against 26 MRSA and nine MSSA while showing a better anti-MRSA activity of 7.8 µg/mL against three MRSA and three MSSA isolates. 

Although most of the target MRSA and MSSA strains (26 out of 41 isolates) used in this study were from Malaysian hospitals, **MFM514** was active against four ATCC *S. aureus* isolates (two MRSA [BAA-1556 and BAA-1688] and two MSSA [ATCC 6358 and ATCC 35556]), in which BAA-1556 and BAA-1688 are community-acquired (CA)-MRSA strains. While these types of MRSA strains have the capacity to infect healthy individuals outside of the hospital and healthcare setting, it has combined methicillin resistance with enhanced virulence and fitness [15].

### 2.3. Results of Cytotoxicity Test and Selectivity Index (SI) Values

To determine the safety of **MFM514** against mammalian cells, we tested the active compound for an in vitro cytotoxicity evaluation. As exhibited in Table 4, **MFM514** did not display any significant toxicity activity against all three normal mammalian cell lines (Vero, WRL-68 and 3T3) with an IC_50_ value of > 625 µg/mL. On the other hand, paclitaxel (a highly toxic anti-cancer compound) showed very low IC_50_ values from 0.0027 to 0.012 µg/mL against all three cell lines. 

A SI value of 40.1 was obtained by dividing the IC_50_ value with a MIC value of 15.6 µg/mL, while a higher SI value of 80.1 could be acquired if a MIC value of 7.8 µg/mL was used. Previous studies have suggested that only compounds with SI > 10 are suitable candidates for further evaluation in animal studies [16,17]. These results also showed concentrations needed to produce inhibitory activity against MRSA/MSSA isolates (MIC = 7.8 to 15.6 µg/mL) were well below the cytotoxic effect (IC_50_ > 625 µg/mL). 

Previous anti-MRSA studies showed lower MIC values but higher cytotoxicity against similar cell lines as compared to **MFM514** [18,19]. On another note, the utilization of skin fibroblast cells (3T3 cells) against **MFM514** was important since the use of different cell lines (kidney and liver cells [representing internal organs] as compared to skin cells [external organ]) may produce a different cytotoxic effect.

### 2.4. Results of Oral Acute Toxicity Test

Based on the high and good SI values, further toxicity test was used to determine the safety of **MFM514** in an animal model. As seen in Table 5, mice administered with **MFM514** did not show a significant difference in the mice’s body weight at days 3, 7, 10 and 14 when compared to the untreated control group. Similarly, mice provided with either control (5% Tween 80) or **MFM514** at 2000 mg/kg did not show any adverse effects or clinical signs of toxicity during the 14 days of the experiment. 

Except for a significant increase in platelet level of mice treated with **MFM514**, there were no significant differences in the haematological and biochemical parameters obtained, as displayed in Tables S1 and S2 (available as Supplementary Materials). Gross macroscopic evaluation of various organs from mice treated with **MFM514** did not demonstrate any abnormal colour or morphological changes as compared to the untreated mice. Following that, there were also no significant differences in the mean relative organ weight of both untreated and treated mice, as exhibited in Table S3 (available as Supplementary Materials).

Based on the Globally Harmonized System of Classification and Labeling of Chemicals (GHS) scheme, the estimated mean lethal dose (LD_50_) for **MFM514** was categorized in Category 5 (>2000 mg/kg < 5000 mg/kg) [20]. Antibiotics such as natamycin, ofloxacin and amikacin have similar GHS Category 5 classification as **MFM514** [21,22]. Previous studies have also reported an increased platelet level in the biochemistry evaluations in treated animals, while no toxicological effect was detected via relative weight gain and gross macroscopic examinations of organs [23,24].

### 2.5. Mice Systemic Infection Test and Estimation of Mean Effective Dose (ED_50_)

Finally, to determine the efficacy of **MFM514** as an anti-MRSA agent, a systemic infection challenge using an animal model was devised. As exhibited in Table 6, mice challenged with MRSA infection and treated with **MFM514** via oral administration at the maximum dose of 125 mg/kg showed significant survival rates of 87.5%, while MRSA-infected mice treated with 25 mg/kg linezolid showed a 100% mice survival. 

No mortalities were observed in healthy/non-treated mice and mice administered with MHB and 5% mucin only (as adjuvant). In contrast, 100% mortality rate was detected in mice infected with MRSA adjuvant and no treatment with **MFM514** or linezolid. Based on the survival rate of **MFM514**, the ED_50_ value was calculated at 29.39 mg/kg. 

Previously, the ED_50_ value of linezolid against MRSA was reported at 15.6 mg/kg [25], which was lower than **MFM514** (ED_50_ = 29.39 mg/kg). Nevertheless, there were other published reports on compounds that have higher/similar ED_50_ values as **MFM514** but were still considered as potential anti-MRSA compounds, such as a new oxadiazole compound (ED_50_ = 44 mg/kg), AFN-1252 (ED_50_ = 29.4 mg/kg) and the new fluoroquinolone anti-MRSA drug, zabofloxacin (ED_50_ = 29.05 mg/kg) [26,27]. 

Currently, AFN-1252 has undergone a phase 2 clinical trial and repackaged as a prodrug, afabicin [28]. Zabofloxacin has been approved for clinical use in the Republic of Korea, the Middle East and North-African countries [29]. On another note, **MFM514** has a drug potential index of less than 10 (ED_50_/MIC = 29.39/15.6 = 1.88), which is the typical ratio for clinically useful antibiotics against MRSA, such as vancomycin and teicoplanin [30].

## 3. Discussion

Our one-pot synthesis protocol follows the green chemistry initiative and has an eco-friendly production approach. **MFM514** are produced using a one-step reaction that utilizes fewer solvents, and less chemical waste was produced for the environment as compared to conventional multi-step reactions. Additionally, **MFM514** is easier to be synthesized since it has a less complex structure as compared to the other current MRSA antibiotics, such as vancomycin and linezolid. Technically, **MFM514** can easily be prepared on a multigram scale in a relatively short time and at a moderate 60% yield, which makes **MFM514** an economically attractive compound to be developed further as a new antibiotic.

The fact that **MFM514** portrayed a five-membered carbon ring as opposed to the conventional four-carbon ring of β-lactam might give way to a new class of antibiotic against MRSA infections. Since **MFM514** is only active against *S. aureus* isolates, it could be determined that **MFM514** has a narrow-spectrum activity. Previous studies showed that narrow-spectrum antibiotics are more favourable as compared to broad-spectrum antibiotics since this type of drug would be less likely to develop antimicrobial resistance and kill ‘good’ bacteria in the human body [31].

In many developing countries, linezolid is the only last-resort and commercially available oral antibiotic against MRSA [2,7]. Oral antibiotics have many advantages over intravenous drugs, such as the absence of cannula-related infections, a lower drug cost and the need for a health professional and equipment to administer intravenous antibiotics [32]. 

In this study, **MFM514** has been able to exert its inhibitory activity while it was delivered via oral administration in the infected mice model. This result would suggest that **MFM514** had survived the liver metabolism mechanism, degradation by the digestive enzymes and acid in the stomach, and interference of absorption by digestive substance of the treated mice [33]. While further pharmaceutical and pharmacodynamic experiments have to be carried out, **MFM514** has the potential as an oral antibiotic candidate. 

## 4. Materials and Methods

### 4.1. Materials

All reagents and solvents were purchased from Merck (Darmstadt, Germany) and Acros Organics (New Jersey, US) and used without further purification. Flash chromatography was performed using silica gel with 200–300 mesh produced by Merck. All reactions and processes of flash chromatography were monitored by the TLC method using silica gel plates with fluorescence F254 and iodine visualization. The melting points were determined with Stuart melting point apparatus SMP30 and uncorrected. Fourier-transformed infrared absorption spectra of both solid and liquid samples were analysed with NICOLET 6700 FT-IR using diamond with ATR. 

Microanalyses were performed on Flash Elemental Analyzer 110 series. The ^1^H NMR and ^13^C NMR spectra were recorded on a Joel- 400 spectrometer at 400 MHz at 125 MHz using CDCl_3_ and DMSO-*d*_6_ as solvents and TMS as internal standard. Coupling constants (J) are expressed in hertz (Hz). Chemical shifts (δ) are given in parts per million (ppm). All target compounds have purity over 95%.

All ATCC bacterial strains (BAA-1556, BAA-1688, ATCC 6358, ATCC 35556, ATCC 25923 and ATCC 33591) and the three mammalian cell lines (WRL-68, Vero CCL-81 and BALB/3T3) were purchased from the American Type Culture Collection (ATCC, Manassas, VA, USA). The additional 37 *S. aureus* clinical isolates were obtained from three local Hospitals in Peninsula Malaysia. 

### 4.2. Synthesis of One-Pot Gamma-Lactam ***1a–1l***: See References [10,11,12]

An equimolar amount of sodium diethyl oxalacetate (47.62 mmol), 40% methylamine in water (47.62 mmol) and 37% formaldehyde solution (47.62 mmol) were heated under reflux in 100 mL EtOH for 1 h. After cooling, the mixture was poured into ice-cooled water and acidified with concentrated hydrochloric acid. The precipitate obtained was filtered out, washed with water and diethyl ether to afford **1a**. Compounds **1b**–**1l** were prepared by the same method.

**4-Hydroxy-1-methyl-5-oxo-2,5- dihydro-1*H*-pyrrole-3-carboxylate** (**1a**): yellowish solid product (40%). m.p. 144–146 ^○^C. IR *ṽ* cm^−^^1^ 3400 (-OH), 1780 (C=O, ester), 1648 (C=C), 1274 (C-N), 734; ^1^H-NMR (400 MHz, CDCl_3_): δ 1.33 (3H, t, J = 7.2 Hz, CH_3_), 3.07 (3H, s, NCH_3_), 3.96 (2H, s, CH_2_), 4.31 (2H, q, J = 7.2 Hz, OCH_2_); 13C-NMR (100 MHz, CDCl_3_): δ 14.30 (CH_3_), 30.10 (NCH_3_), 48.10 (CH_2_), 61.20 (OCH_2_), 107.60 (quat. C), 157.40 (C=O), 164.10 (C=O), 165.20 (COH); Anal. Calcd. for C_8_H_11_NO_4_ (185.07): C, 51.89; H, 5.99; N, 7.56; O, 34.56. Found: C, 51.80; H, 4.65; N, 7.70; O, 35.85. GCMS *m*/*z* (EI, + ve): found 185.0 ([M]^+^), C_8_H_11_NO_4_ calculated 185.07. 

**Ethyl 4-hydroxy-1,2-dimethyl-5-oxo-2,5- dihydro-1*H*-pyrrole-3-carboxylate** (**1b**): light yellow solid product (62%). m.p.101–103 ^○^C. IR *ṽ* cm^−1^: 3094 (-OH), 2980 (NH, amide), 1706 (C=O, ester), 1694 (C=C), 1657 (N-C=O, amide), 1461 (CH_2_), 1386 (CH_3_), 1296 (C-N); ^1^H-NMR (400 MHz, CDCl_3_): δ 1.34 (t, J = 7.3 Hz, 3H), 1.40 (d, J = 6.4 Hz, 3H), 3.02 (d, J = 14.6 Hz, 3H), 4.08 (q, J = 6.6 Hz, 1H), 4.39–4.25 (m, 2H), ^13^C-NMR (100 MHz, CDCl_3_): δ 14.35 (CH_3_), 17.21 (CH_3_), 27.21 (NCH_3_), 54.42 (CH), 61.30 (OCH_2_), 112.97 (quat. C), 157.96 (C=O), 163.26 (C=O), 165.66 (COH). GCMS *m*/*z* (EI, + ve): found 199.10 ([M]^+^), C_9_H_13_NO_4_ calculated 199.08.

### 4.3. Synthesis of Decarboxylated ***2a***,***2b*** and Hydrazone Derivative ***3a**–**3d***

The results for this compounds have already published in Refs. [10,11,12]. 

### 4.4. Synthesis of One-Pot Product ***4a**–**4d***

A mixture of sodium diethyl oxaloacetate salt (142.73 mmol), 37% formaldehyde (142.73 mmol) and 25% ammonia (214.10 mmol) in ethanol was refluxed towards completion (0.5–2 h). Iced water was added to the mixture after cooling, and HCl was then added dropwise to pH 1. The solid product was filtered upon appearance. Traces of aldehyde in the crude product was washed with water and ether to afford **4a**. Compounds **4b**–**4d** were prepared by the same method.

**Ethyl 4-hydroxy-5-oxo-2,5-dihydro-1*H*-pyrrole-3-carboxylate** (**4a**): white solid product (60%). m.p. 106–109 ^○^C. IR *ṽ* cm^−^^1^: 3344 (-OH), 2986 (NH, amide), 1782 (C=O, ester), 1687 (C=C), 1670 (-N-C=O, amide), 1302 (C-N); ^1^H-NMR (400 MHz, CDCl_3_): δ 4.91–4.85 (2H, s, CH_2_), 4.39–4.32 (2H, q, J = 7.2 Hz, CH_2_), 1.38–1.31 (3H, t, J = 7.1 Hz, CH_3_); ^13^C-NMR (100 MHz, CDCl_3_): δ 166.54 (COH), 164.23 (C=O) 151.29 (C=O), 116.09 (quat. C), 66.26 (OCH_2_), 62.09 (CH_2_), 14.34 (CH_3_); Anal. Calcd. for C_7_H_9_NO_4_: C, 49.12; H, 5.30; N, 8.18; O, 37.39. Found: C, 49.30; H, 4.65; N, 7.04; O, 39.01; GCMS *m*/*z* (EI, +ve): found 172.00 ([M]^+^), C_7_H_9_NO_4_ calculated 172.06.

**Ethyl 4-hydroxy-1-(2-hydroxyethyl)-5-oxo-2,5-dihydro-1*H*-pyrrole-3-carboxylate** (**4b**): light orange solid product (35%). m.p. 132–134 ^○^C. IR *ṽ* cm^−1^: 3479 (-CH_2_OH), 3310 (-CH-OH), 2920 (NH, amide), 1694 (C=O, ester), 1655 (C=C), 1513 (N-C=O); ^1^H-NMR (400 MHz, CD_3_OD): δ 4.26 (q, J = 7.2 Hz, 2H), 4.13 (s, 2H), 3.76–3.68 (m, 2H), 3.62–3.51 (m, 2H), 1.29 (t, J = 7.1 Hz, 3H); ^13^C-NMR (100 MHz, CD_3_OD): δ 176.98 (COH), 165.88 (C=O), 163.69 (C=O), 107.90 (quat. C), 60.32 (OCH_2_), 59.42 (CH_2_OH), 48.30 (CH_2_-N), 45.32 (CH_2_), 13.27 (CH_3_); Anal.Calcd. for C_9_H_13_NO_5_; C, 50.23; H, 6.09; N, 6.51; O, 37.17. Found: C, 48.98; H, 5.91; N, 5.87; O, 39.24; GCMS *m*/*z* (EI, +ve): found 215.00 ([M]^+^), C_9_H_13_NO_5_ calculated 215.08.

**Ethyl 1-butyl-4-hydroxy-5-oxo-2,5-dihydro-1*H*-pyrrole-3-carboxylate** (**4c**): white solid product (25%). m.p. 109–111 °C. IR *ṽ* cm^−1^: 3099 (-OH), 2956 (NH, amide), 1663 (C=O, ester), 1447 (CH_2_), 1362 (CH_3_); ^1^H-NMR (400 MHz, CDCl_3_): δ 4.30 (q, J = 7.2 Hz, 2H), 3.97 (t, J = 15.8 Hz, 2H), 3.48 (t, J = 7.5 Hz, 2H), 1.57 (s, 2H), 1.32 (t, J = 7.3 Hz, 5H), 0.95–0.88 (m, 3H); ^13^C-NMR (100 MHz, CDCl_3_): δ 165.09 (COH), 164.24 (C=O), 156.80 (C=O), 107.68 (quat. C), 61.15 (OCH2), 46.24 (CH_2_-N), 42.89 (CH_2_), 30.35 (CH_2_), 19.99 (CH_2_), 14.35 (CH_3_), 13.75 (CH_3_); Anal. Calcd. for C_11_H_17_NO_4_; C, 58.14; H, 7.54; N, 6.16; O, 28.16. Found: C, 53.85; H, 7.04; N, 5.06; O, 34.05; GCMS *m*/*z* (EI, +ve): found 227.10 ([M]^+^), C_11_H_17_NO_4_ calculated 227.12.

**Ethyl 4-hydroxy-1-(4-hydroxyphenyl)-5-oxo-2,5-dihydro-1*H*-pyrrole-3-carboxylate** (**4d**): brownish yellow solid product (5%). m.p. > 160 ^○^C decomposed. IR *ṽ* cm^−1^: 3235 (-OH), 2990 (NH, amide), 1654 (C=O, ester), 1595 (C=C), 1514 (N-C=O), 757 (Ar-OH); ^1^H-NMR (400 MHz, CD_3_OD): δ 7.49 (d, J = 8.7 Hz, 2H), 6.80 (d, J = 9.1 Hz, 2H), 4.40 (s, 2H), 4.28 (d, J = 6.9 Hz, 2H), 1.31 (t, J = 7.1 Hz, 3H); ^13^C-NMR (100 MHz, CD_3_OD): δ 169.17 (COH), 166.68 (C=O), 161.33 (C=O), 155.31 (quat. C), 130.39 (quat. C), 121.87 (CH-Ar), 115.28 (CH-Ar), 110.34 (quat. C), 60.37 (OCH_2_) 47.07 (CH_2_), 13.32 (CH_3_); Anal.Calcd. for C_13_H_13_NO_5_; C, 59.31; H, 4.98; N, 5.32; O, 30.39. Found: C, 51.64; H, 4.92; N, 4.07; O, 29.37; GCMS *m*/*z* (EI, +ve): found 286.90 ([M+Na]^+^), C_13_H_13_NO_5_ calculated 263.08.

### 4.5. Synthesis of Enamine ***4e**–**4k***

A mixture of **4a** (5.84 mmol), aniline (6.43 mmol) and formic acid (9.34 mmol) was refluxed for 24 h. The evaporation of the solvent gave the crude product, which was purified by flash column chromatography on silica gel using hexane:ethyl acetate (9:1) to afford **4e**. Compounds **4f**–**4k** were prepared by the same method.

**Ethyl 5-oxo-4-(phenylamino)-2,5-dihydro-1*H*-pyrrole-3-carboxylate** (**4e**): yellow solid (22%). m.p. 59–62 ^○^C. IR *ṽ* cm^−^^1^: 3396 (NH), 1700 (C=O, ester), 1675 (C=C), 1539 (N-C=O), 758 (NH-Ar); ^1^H-NMR (400 MHz, CDCl_3_): δ 7.30 (t, 2H), 7.14 (t, J = 7.5 Hz, 1H), 7.08 (d, J = 7.3 Hz, 2H), 4.93 (s, 2H), 4.23 (q, J = 7.2 Hz, 2H), 1.25 (t, J = 7.1 Hz, 3H); ^13^C-NMR (100 MHz, CDCl_3_) 167.51 (C=O), 163.89 (C=O), 137.85 (quat. C), 137.61 (aromatic. C), 128.72 (CH-Ar), 125.10 (CH-Ar), 122.73 (CH-Ar), 111.83, (quat. C), 67.74 (OCH_2_), 60.94 (CH_2_), 14.28 (CH_3_); LCMS m/z (ESI-QTOF, +ve): found 248.0916 ([M+2H]^+^), C_13_H_14_N_2_O_3_ calculated 246.099.

**Ethyl 4-((4-ethylphenyl)amino)-5-oxo-2,5-dihydro-1*H*-pyrrole-3-carboxylate** (**4f**): brown oily product (36%). IR *ṽ* cm^−^^1^: 3328 (NH), 1766 (C=O, ester), 1685 (C=C), 1517 (N-C=O), 1459 (CH_2_), 1356 (_CH3)_, 759 (NH-Aro-CH_2_CH_3_); ^1^H-NMR (400 MHz, CDCl_3_): δ 7.13 (d, J = 8.5 Hz, 2H), 7.01 (d, J = 6.2 Hz, 2H), 4.93 (s, 2H), 4.23 (q, J = 7.2 Hz, 2H), 2.62 (q, J = 7.6 Hz, 2H), 1.28–1.19 (m, 6H); ^13^C-NMR (100 MHz, CDCl_3_) δ 141.35 (C=O), 137.92 (C=O), 135.36 (quat. C), 128.12 (CH-Ar), 122.98 (CH-Ar), 119.530 (aromatic. C), 113.79 (aromatic. C), 110.92 (quat. C), 60.82 (OCH_2_), 28.39 (CH_2_), 15.59 (CH_3_), 14.29 (CH_3_); LCMS *m*/*z* (ESI-QTOF, +ve): found 276.1282 ([M+2H]^+^), C_15_H_18_N_2_O_3_ calculated 274.1312.

**Ethyl 4-((4-methoxyphenyl) amino)-5-oxo-2,5-dihydro-1*H*-pyrrole-3-carboxylate** (**4g**): light-yellow solid product (25%). m.p. 70–71 ^○^C. IR *ṽ* cm^−^^1^: 3327 (NH), 2982 (NH, amide), 1766 (C=O, ester), 1638 (C=C), 1517 (N-C=O), 755 (NH-Ar-OMe); ^1^H-NMR (400 MHz, CD_3_COCD_3_) 8.79 (d, J = 10.9 Hz, 2H), 8.54 (d, J = 11.2 Hz, 2H), 6.14 (s, 2H), 5.30 (q, J = 8.9 Hz, 2H), 4.73 (s, 3H), 1.59 (t, J = 8.9 Hz, 3H); ^13^C-NMR (100 MHz, CD_3_COCD_3_) δ 178.90 (C=O), 166.36 (C=O), 159.38 (aromatic. C), 157.52 (quat. C), 143.66 (aromatic. C), 130.63 (quat. C), 125.11 (CH-Ar), 113.93 (quat. C), 67.56 (OCH_2_), 60.79 (CH_2_), 55.54 (OCH3), 14.35 (CH_3_); LCMS *m*/*z* (ESI-QTOF, +ve): found 278.0824 ([M+2H]^+^), C_14_H_16_N_2_O_4_ calculated 276.1104.

**Ethyl 4-((4-hydroxyphenyl)amino)-5-oxo-2,5-dihydro-1*H*-pyrrole-3-carboxylate** (**4h**): yellow solid product (19%). m.p. 70–71 ^○^C. IR *ṽ* cm^−^^1^: 3322 (-OH), 2948 (NH-Ar) 2836 (NH, amide), 1650 (C=O, ester), 1449 (C=C), 1441 (N-C=O), 1013 (Ar-OH); ^1^H-NMR (400 MHz, CD_3_OD) δ 6.92 (dd, J = 6.6, 2.1 Hz, 2H), 6.68 (dd, J = 6.9, 1.8 Hz, 2H), 4.89 (s, 2H), 4.12 (q, J = 7.2 Hz, 2H), 1.15 (t, J = 7.3 Hz, 3H), ^13^C-NMR (100 MHz, CD_3_OD) δ 174.43 (C=O), 173.31 (C=O), 150.16 (quat. C ArOH), 146.00 (quat. CNH), 145.19 (aromatic. C), 119.90 (CH-Ar), 119.72 (CH-Ar), 115.79 (quat. C), 60.79 (OCH_2_), 29.71 (CH_2_), 14.29 (CH_3_). GCMS *m*/*z* (EI, + ve): found 263.10 ([M + H]^+^), C_13_H_14_N_2_O_4_ calculated 262.10.

**Ethyl 4-(naphthalen-1-ylamino)-5-oxo-2,5-dihydro-1*H*-pyrrole-3-carboxylate** (**4i**): light-yellow solid (18%). m.p. 112–113 ^○^C. IR *ṽ* cm^−^^1^: 3304 (-OH), 3049 (NH, amide), 1768 (C=O, ester), 1685 (C=C), 1629 (N-C=O), 750 (Ar-Napthyl); ^1^H-NMR (400 MHz, CDCl_3_) δ 7.82–7.71 (m, 3H), 7.54–7.36 (m, 3H), 7.28–7.22 (m, 1H), 4.98 (s, 2H), 4.24 (q, J = 7.2 Hz, 2H), 1.26–1.20 (m, 3H), ^13^C NMR (100 MHz, CDCl_3_) δ 167.54 (C=O), 163.95 (C=O), 137.63 (quat. CNH), 135.41 (Aromatic. C), 133.54 (Aromatic. C), 131.13 (Aromatic. C), 128.49 (CH-Ar), 127.78 (CH-Ar), 127.47 (CH-Ar), 126.67 (CH-Ar), 125.41 (CH-Ar), 122.40 (CH-Ar), 119.38 (CH-Ar), 112.28 (quat. C), 67.80 (OCH2), 61.00 (CH_2_), 14.26 (CH_3_). GCMS *m*/*z* (EI, +ve): found 297.12 ([M + H]^+^), C_17_H_16_N_2_O_3_ calculated 296.12.

**Ethyl 5-oxo-4-(2-tosylhydrazineyl)-2,5-dihydro-1*H*-pyrrole-3-carboxylate** (**4j**): light-yellow solid product (49%). m.p. 164–165 °C. IR *ṽ* cm^−^^1^ 3295 (-OH), 2996 (NH, amide), 1763 (C=O, ester), 1693 (C=C), 1663 (N-C=O), 1338 (S=O), 761 (Ar-CH_3_); ^1^H-NMR (400 MHz, CDCl_3_) δ 7.72 (d, J = 8.2 Hz, 2H), 7.25 (d, J = 8.2 Hz, 2H), 4.50 (s, 2H), 4.24 (q, J = 7.2 Hz, 2H), 2.41 (s, 3H), 1.30 (t, J = 7.1 Hz, 3H); ^13^C-NMR (100 MHz, CDCl_3_) δ 175.46 (C=O), 167.91 (C=O), 163.32 (quat. CNH), 149.59 (Aromatic. C), 143.99 (Aromatic. C), 129.45 (CH-Ar), 128.43 (CH-Ar), 115.85 (quat. C), 66.69 (OCH_2_), 61.56 (CH_2_), 21.69 (Ar-CH_3_), 14.28 (CH_3_). GCMS *m*/*z* (EI, + ve): found 341.0 ([M + 2H]^+^), C_14_H_17_N_3_O_5_S calculated 339.09.

**Ethyl 1-(2-hydroxyethyl)-4-((4-methoxyphenyl)amino)-5-oxo-2,5-dihydro-1*H*-pyrrole-3-carboxylate** (**4k**): brown solid product (79%). m.p. 79–81 °C. IR *ṽ* cm^−1^: 3464 (-OH), 3310 (NH-Ar), 2922 (NH, amide), 1694 (C=O, ester), 1591 (C=C), 1513 (N-C=O), 778 (Ar-OMe); ^1^H-NMR (400 MHz, CDCl_3_) δ 8.03 (s, 1H), 7.03 (d, J = 9.1 Hz, 2H), 6.81 (d, J = 6.9 Hz, 2H), 4.16 (q, J = 7.5 Hz, 4H), 3.82- 3.73 (m, 5H), 3.59 (t, J = 5.0 Hz, 2H), 2.35 (s, 1H), 1.26–1.14 (m, 3H); ^13^C-NMR (100 MHz, CDCl_3_) δ 166.08 (C=O), 165.07 (C=O), 157.07 (Aromatic. COMe), 143.97 (quat. CNH), 131.65 (Aromatic. CNH), 124.86 (CH-Ar), 113.86 (CH-Ar), 102.82 (quat. C), 61.31 (OCH_2_), 60.16 (CH_2_OH), 55.54 (OCH_3_), 49.20 (CH_2_NH), 46.51 (CH_2_), 14.40 (CH_3_). GCMS *m*/*z* (EI, + ve): found 320.10 ([M]^+^), C_16_H_20_N_2_O_5_ calculated 320.14.

### 4.6. Synthesis of Reduced Gamma-Lactam ***5a, 5b***: See Reference [13]

**Ethyl 4-hydroxy-5-oxopyrrolidine-3-carboxylate** (**5a**): colourless oily product (58%). IR *ṽ* cm^−^^1^: 3392 (OH), 2915 (NH, amide), 1779 (C=O, ester), 1470 (CH_2_), 1352 (CH_3_); ^1^H-NMR (400 MHz, CDCl_3_) δ 4.62 (d, J = 7.8 Hz, 1H), 4.55 (d, J = 12.0 Hz, 1H), 4.44–4.25 (m, 1H), 4.23 (t, J = 10.7 Hz, 2H), 3.65–3.25 (m, 1H), 1.28 (t, J = 7.1 Hz, 3H; ^13^C-NMR (100 MHz, CDCl_3_) δ 154.58 (C=O), 145.25 (C=O), 68.38 (CHOH), 66.39 (OCH_2_), 62.10 (CH_2_), 45.61 (CH), 14.17 (CH_3_); GCMS *m*/*z* (EI, + ve): found 174.00 ([M + H]^+^), C_7_H_11_NO_4_ calculated 173.07.

**Ethyl 4-hydroxy-1-methyl-5-oxopyrrolidine-3-carboxylate** (**5b**): white solid product (98%). m.p. 116–118 ^○^C. IR *ṽ* cm^−^^1^: 3217 (-OH), 2989 (NH, amide), 1721.73 (C=O, ester), 1503 (N-C=O), 1463 (CH_2_), 1353 (CH_3_); ^1^H-NMR (400 MHz, CD_3_OD): δ 4.44 (d, J = 7.8 Hz, 1H), 4.15 (q, J = 7.2 Hz, 2H), 3.64 (dd, J = 10.1, 3.2 Hz, 1H), 3.28 (t, J = 1.6 Hz, 1H), 3.05 (s, 1H), 2.84 (d, J = 5.5 Hz, 3H), 1.28–1.20 (m, 3H); ^13^C-NMR (100 MHz, CD_3_OD): δ 172.93 (C=O), 170.711 (C=O), 70.27 (CHOH), 60.71 (OCH_2_), 47.88 (CH_2_), 43.46 (CH), 28.70 (CH_3_-N), 13.15 (CH_3_); GCMS *m*/*z* (EI, + ve): found 187.10 ([M]^+^), C_8_H_13_NO_4_ calculated 187.0.

### 4.7. Antibacterial Activity Studies

All *Staphylococcus aureus* isolates were maintained in Protect Bacterial Preservers (Technical Service Consultants Limited, Lancashire, UK) at −20 °C. Prior to use, isolates were subcultured overnight at 37 °C in Mueller–Hinton broth (MHB), adjusted to obtain turbidity comparable to that of 0.5 McFarland standard (10^8^ CFU/mL) using a cell density meter (Biochrom WPA CO8000, Cambridge, UK) between absorbance of 0.08 and 0.1 at 600 nm. Serial two-fold dilutions of synthesized compounds dissolved in dimethyl sulfoxide (DMSO) were prepared prior to addition of 100 µL of standardized *S. aureus* culture followed by incubation at 37 °C for 24 h. 

The MIC value was defined as the lowest concentration producing no visible growth (absence of turbidity and/or precipitation) as observed through naked eye. For further reconfirmation, 20 µL (20 mg/mL) of 3-(4,5-dimethylthiazol-2-yl)-2,5-diphenyltetrazolium bromide (MTT) reagent was added to the bacterial suspension in the selected wells, followed by 20 min of incubation at 37 °C. The reagent/bacterial suspension colour will remain clear/yellowish for inhibitory activity as opposed to dark blue indicating growth.

### 4.8. Cytotoxicity Studies

The cytotoxicity of **MFM514** was evaluated using the MTT assay as described previously [34]. The following three normal mammalian cell lines: WRL-68, Vero and 3T3, representing liver, kidney and skin fibroblast, respectively, were used in this study. Cells were cultured in Dulbecco’s modified eagle medium (DMEM) and supplemented with 5% foetal bovine serum and 1% penicillin-streptomycin. 

Cells were allowed to attach and spread overnight prior to 72 h of incubation with **MFM514** at various concentrations. MTT assay was carried out to determine the number of viable cells relative to the control. Paclitaxel was used as the positive control. IC_50_ values were determined from the corresponding dose-response curve. SI values were determined via IC_50_/MIC.

### 4.9. Oral Acute Toxicity Studies

The fixed-dose procedure for oral acute toxicity was employed as recommended by the Organization for Economic Co-operation and Development (OECD) [35]. Five mice per group were orally administered with a fixed recommended dose of 2000 mg/kg body weight of **MFM514** following a period of fasting. Observations for toxicity signs were conducted at 30 min, 4h and daily up to 14 days/dose. The mean lethal dose (LD_50_) value was estimated based on the GHS [20]. At the end of the experiment, blood was collected from untreated (control) and treated mice through cardiac puncture procedure in EDTA-coated tubes for both haematology and biochemistry analysis on the 14th day following 12 h fasting period. 

The haematological evaluations were determined using a Mindray BC-2800Vet Auto Hematology Analyzer (Mindray Corporation, Shenzhen, China). Consequently, the same whole blood-containing tubes was centrifuged at 4000 rpm and the supernatant was collected and introduced into new tubes for the subsequent biochemical analysis using reagent kits and a Roche Cobas C111 Clinical Chemistry Analyzer (Indianapolis, IN, USA). Lastly, the mean relative weight of each organ to its respective body weight and macroscopic evaluation of each organ from both treated and untreated mice were compared.

### 4.10. Mice Systemic Infection Assay

This study was performed as described previously with minor modifications [25]. A group of six ICR mice was given an MRSA adjuvant via intraperitoneal (i.p.) route. An MRSA adjuvant consisted of a standardized 1.2 × 10^9^ CFU/mL MRSA culture suspended in equal volume of 5% mucin. **MFM514** was prepared in 5% Tween 80 and dissolved into four serial concentrations between 15.6 mg/kg and 125 mg/kg. 

Subsequently, **MFM514** was administered in single dose via oral route (p.o.) one hour after i.p. infection. Since **MFM514** was delivered using the oral route, linezolid (a commercially available oral antibiotic against MRSA infection) was selected as the positive control drug. Survivability of the mice was observed over seven days. The total number of survivors at each dose was used to calculate the ED_50_ value.

## 5. Patents

Mohd Fazli Mohammat, Ahmad Sazali Hamzah, Zurina Shaameri, Sharifah Aminah Syed Mohamad, Saiful Azmi Johari and Mastura Mohtar. An inhibitor for inhibiting methicillin-resistance *Staphylococcus aureus* (MRSA) activity. PI2017704143.

## Figures and Tables

**Table 1:** List of gamma-lactam structures synthesized in this study; **1a**–**5b**.

Code	Structure	Code	Structure	Code	Structure
**1a**	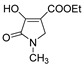	**MFM514**	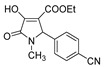	**4d**	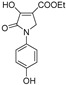
**1b**	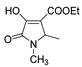	**1l**	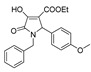	**4e**	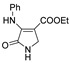
**1c**	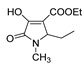	**2a**	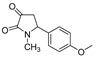	**4f**	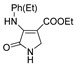
**1d**	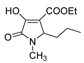	**2b**	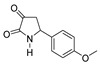	**4g**	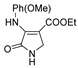
**1e**	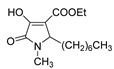	**3a**	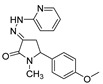	**4h**	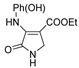
**1f**	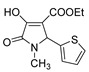	**3b**	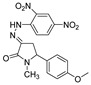	**4i**	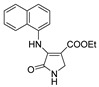
**1g**	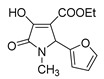	**3c**	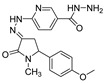	**4j**	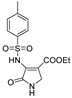
**1h**	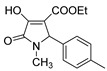	**3d**	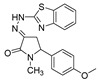	**4k**	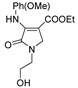
**1i**	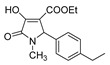	**4a**	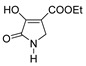	**5a**	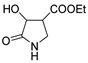
**1j**	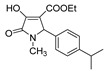	**4b**	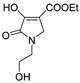	**5b**	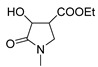
**1k**	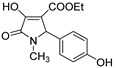	**4c**	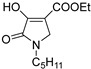		

**Table 2:** Initial minimum inhibitory concentration (MIC) values of 32 gamma-lactam compounds against MRSA and MSSA isolates.

Compounds	MIC Values (µg/mL)	
	MRSA (ATCC 33591)	MSSA (ATCC 25923)
**1a–1d**	>500	>500
**1e**	125	250
**1f–1l**	>500	>500
**MFM514**	15.6	31.3
**2a–5b**	>500	>500

**Table 3:** MIC values of **MFM514** against additional 41 *S. aureus* isolates.

*S. aureus* Isolates	MIC Values (µg/mL)
**MRSA isolates**	
A1, A2, A3, A4, A7, A8, BAA-1556, C1, C4, C5, C8, HN1, HN3, HN4, HN5, HN13, HN14, HS3178, HS770, HS3175, N441, N391, N829, N850, N1406, U949	15.6
BAA-1688, D3, HN7	7.8
**MSSA isolates**	
B1, UM9, ATCC 6538, HN6, HN11, A5, A6, C6, ATCC 35556	15.6
HN8, HN9, HN10	7.8

**Table 4:** Mean inhibitory concentration (IC_50_) of **MFM514** against three mammalian cell lines and determination of selectivity index (SI) values.

Compounds	Cell Lines	IC_50_	MIC	SI Values (IC_50_/MIC)
		(µg/mL)		
**MFM514**	3T3, Vero and WRL-68	>625	7.8	80.1
			15.6	40.1
Paclitaxel	3T3	0.012 ± 0.01	ND ^1^	ND
	Vero	0.0055 ± 0.02		
	WRL-68	0.0027 ± 0.06		

^1^ ND = Not determined.

**Table 5:** Body weight of mice receiving **MFM514** at a single dose of 2000 mg/kg.

Mice Group	Cell Lines					% of Weight Change
	Day 0	Day 3	Day 7	Day 10	Day 14	
Untreated (5% Tween 80)	26.3 ± 1.2	27.2 ± 1.7	27.8 ± 1.4	28.2 ± 1.8	29.5 ± 1.2	11.0 ± 3.2
**MFM514**	26.4 ± 1.6	27.9 ± 3.0	28.8 ± 3.1	28.5 ± 1.9	29.7 ± 2.3	11.1 ± 2.3

Values were expressed as mean ± standard deviation (SD) of five mice. *p* < 0.05 was considered statistically significant difference.

**Table 6:** Survival rates of untreated and MRSA-infected mice after treated with **MFM514**.

Mice Groups	Untreated/Treated Mice	Total of Mice Survive	% Survive
Untreated mice	MRSA adjuvant only	0/8	0
	MRSA adjuvant + 25 mg/kg linezolid	8/8	100
	MHB + 5% mucin only	8/8	100
	Healthy and untreated mice	8/8	100
Mice treated with **MFM514**	125 mg/kg	7/8	87.5
	62.5 mg/kg	5/8	62.5
	31.3 mg/kg	4/8	50
	15.6 mg/kg	3/8	37.5

## Data Availability

The data presented in this study are available in Supplementary Materials.

## Supplementary Materials

The following supporting information can be downloaded at: https://www.mdpi.com/article/10.3390/molecules28062575/s1, Table S1: Hematological analysis of untreated and treated mice with **MFM514**; Table S2. Biochemistry analysis of untreated and treated mice with **MFM514**; Table S3. Biochemistry analysis of untreated and treated mice with **MFM514**.
