# Supplementary material for: Investigating the Antibacterial Effects of Synthetic Gamma-Lactam Heterocycles on Methicillin-Resistant Staphylococcus aureus Strains and Assessing the Safety and Effectiveness of Lead Compound MFM514
Source: Molecules. 2023 Mar 12;28(6):2575. doi: 10.3390/molecules28062575 (PMC10058495; doi:10.3390/molecules28062575)
Supplement: Supplementary file 1 [file molecules-28-02575-s001.zip › molecules-2265761-supplementary.pdf]

Supplementary materials

Investigating the Antibacterial Effects of Synthetic Gamma-Lactam Heterocycles on Methicillin-Resistant *Staphylococcus aureus* Strains and Assessing the Safety and Effectiveness of Lead Compound MFM514

Saiful Azmi Johari [1], Mastura Mohtar [1], Mohd Fazli Mohammat [2]*, Fatin Nur Ain Abdul Rashid [2], Muhamad Zulfaqar Bacho [2], Azman Mohamed [1], Mohamad Jemain Mohamad Ridhwan [3], Sharifah Aminah Syed Mohamad [4]

[1] Bioactivity Programme, Natural Products Division, Forest Research Institute Malaysia (FRIM), 52109 Kepong, Selangor, Malaysia
[2] Organic Synthesis Laboratory, Institute of Science, Universiti Teknologi MARA (UiTM), Puncak Alam Campus, Puncak Alam, Kuala Selangor 42300, Selangor, Malaysia
[3] Pharmacy Programme, Sultan Azlan Shah Allied Health Sciences College, 31250 Tanjung Rambutan, Perak, Malaysia
[4] Faculty of Applied Sciences, Universiti Teknologi MARA (UiTM), 40450 Shah Alam, Selangor, Malaysia.

* Correspondence: mohdfazli@uitm.edu.my

Abstract: Methicillin-resistant Staphylococcus aureus (MRSA) continues to be as one of the main causes of hospital-acquired infections in all regions of the world while linezolid is one of the only commercially-available oral antibiotic available against this dangerous Gram-positive pathogen. In this study, the antibacterial activity from 32 analogues of synthetic gamma-lactam heterocycles against MRSA were determined. Amongst screened analogues for the minimum inhibitory concentration (MIC) assay, compound **MFM514** displayed good inhibitory activity with MIC values of 7.8-15.6 μg/ml against 30 MRSA and 12 methicillin-sensitive S. aureus (MSSA) clinical isolates while cytotoxicity evaluations displayed a mean inhibitory concentration (IC50) value of > 625 μg/ml, displaying a potential to becoming as a lead compound. In subsequent animal studies for **MFM514**, a single-dose oral acute toxicity test revealed an estimated mean lethal dose (LD50) value of < 5000 mg/kg while in the mice infection test, a mean effective dose (ED50) value of 29.39 mg/kg was obtained via oral administration. These results suggests that gamma-lactam carbon-skeleton particularly **MFM514** is highly recommended to be evaluated further as a new safe and efficacious orally delivered antibacterial agent against MRSA.

Keywords: gamma-lactam; antibacterial; oral anti-MRSA agent; acute toxicity; mice infection

Table S1. Haematological analysis of untreated and treated mice with **MFM514**

Haematological values	Untreated (5% Tween 80)	MFM514 at 2000 mg/kg
White blood cells (x 10^9/L)	5.8 ± 1.7	6.5 ± 1.7
Lymphocyte (x 10^9/L)	4.6 ± 1.4	5.1 ± 1.55
Monocyte (x 10^9/L)	0.18 ± 0.08	0.22 ± 0.04
Granulocyte (x 10^9/L)	1.0 ± 0.5	1.1 ± 0.2
Lymphocyte (%)	80.2 ± 5.6	77.7 ± 2.4
Monocyte (%)	3.2 ± 0.7	3.6 ± 0.6
Granulocyte (%)	16.5 ± 5.3	18.8 ± 1.9
Red blood cells (x 10^{12}/L)	8.7 ± 0.4	8.8 ± 0.6
Hemoglobin (g/dL)	14.2 ± 1.3	14.0 ± 0.7
Hematocrit (%)	33.9 ± 16.3	41.7 ± 1.8
Mean corpuscular volume (fL)	47.2 ± 0.6	47.6 ± 1.8
Mean cell hemoglobin (pg)	15.9 ± 0.2	15.6 ± 0.6
Mean cell haemoglobin concentration (g/dL)	33.6 ± 0.6	33.5 ± 0.5
Red blood cell distribution width (%)	16.1 ± 0.7	16.5 ± 0.6
Platelets counts (x 10^9/L)	659.0 ± 72.6	[1]882.6 ± 173.6
Mean platelet volume (fL)	4.4 ± 0.7	4.3 ± 0.2
Platelet distribution width	16.5 ± 0.5	16.4 ± 0.2
Plateletcrit (%)	0.3 ± 0.1	0.4 ± 0.1

[1]a significant value change was detected

Values were expressed as mean ± standard deviation (SD) of five mice. $p<0.05$ was considered statistically significant difference

Table S2. Biochemistry analysis of untreated and treated mice with **MFM514**

**Biochemistry analysis**	**Untreated (5% Tween 80)**	**MFM514 at 2000 mg/kg**
**Liver Profile**		
Total protein (g/L)	68.7 ± 29.6	61.7 ± 6.9
Albumin (g/L)	37.8 ± 15.5	36.9 ± 2.0
Alanine phosphatase (U/L)	124.4 ± 61.7	127.4 ± 16.7
Alanine transaminase (ALT) (U/L)	87.3 ± 39.6	87.3 ± 9.7
Aspartate transaminase (AST) (U/L)	102.5 ± 42.2	95.5 ± 12.8
AST/ALT ratio	1.17	1.09
**Renal Profile**		
Uric acid (μmol/L)	104.1 ± 42.6	104.0 ± 36.9
**Lipid Profile**		
High density lipoprotein (mmol/L)	1.8 ± 0.8	1.9 ± 0.3
Triglycerides (mmol/L)	0.8 ± 0.3	0.9 ± 0.1
Glucose (mmol/L)	10.0 ± 4.2	9.6 ± 1.3

Values were expressed as mean ± standard deviation (SD) of five mice. $p<0.05$ was considered statistically significant difference

Table S3. Biochemistry analysis of untreated and treated mice with **MFM514**

**Organ examined**	**Relative organ weight (g)**	
	**Untreated (5% Tween 80)**	**MFM514 at 2000 mg/kg**
Heart	0.41 ± 0.05	0.43 ± 0.03
Thymus	0.18 ± 0.03	0.16 ± 0.02
Lung	0.72 ± 0.11	0.77 ± 0.15
Liver	4.47 ± 0.65	5.20 ± 0.56
Left kidney	0.46 ± 0.04	0.49 ± 0.05
Right kidney	0.44 ± 0.04	0.47 ± 0.04
Spleen	0.43 ± 0.15	0.44 ± 0.08
Intestine	11.95 ± 1.28	12.28 ±0.79
Left ovary	0.022 ± 0.001	0.033 ± 0.012
Right ovary	0.021 ± 0.001	0.030 ± 0.014
Brain	1.28 ± 0.08	1.34 ± 0.03

Values were expressed as mean ± standard deviation (SD) of five mice. $p<0.05$ was considered statistically significant difference
